# Characteristics and Clinical Outcomes of 116,539 Patients Hospitalized with COVID-19—Poland, March–December 2020

**DOI:** 10.3390/v13081458

**Published:** 2021-07-27

**Authors:** Mariusz Gujski, Mateusz Jankowski, Daniel Rabczenko, Paweł Goryński, Grzegorz Juszczyk

**Affiliations:** 1 Department of Public Health, Medical University of Warsaw, 02-097 Warsaw, Poland; mariusz.gujski@wum.edu.pl (M.G.); grzegorz.juszczyk@wum.edu.pl (G.J.); 2 Centre of Postgraduate Medical Education, School of Public Health, 01-826 Warsaw, Poland; 3 Department of Population Health Monitoring and Analysis, National Institute of Public Health—National Institute of Hygiene, 00-791 Warsaw, Poland; daniel@pzh.gov.pl (D.R.); pawel@pzh.gov.pl (P.G.)

**Keywords:** SARS-CoV-2, COVID-19, risk factors, mortality, hospitalization, intensive care unit, COVID-19 prediction ICU, Poland

## Abstract

The severe acute respiratory syndrome coronavirus 2 (SARS-CoV-2) causes Coronavirus Disease 2019 (COVID-19). This study aimed to characterize patients hospitalized with COVID-19 in Poland between March and December 2020, as well as to identify factors associated with COVID 19–related risk of in-hospital death. This retrospective analysis was based on data from the hospital discharge reports on COVID-19 patients hospitalized in Poland between March and December 2020. A total of 116,539 discharge reports on patients hospitalized with COVID-19 were analyzed. Among patients with COVID-19, 21,490 (18.4%) died during hospitalization. Patients over 60 years of age (OR = 7.74; 95%CI: 7.37–8.12; *p* < 0.001), men (OR = 1.42; 95%CI: 1.38–1.47; *p* < 0.001) as well as those with cardiovascular diseases (OR = 1.51; 95%CI: 1.46–1.56; *p* < 0.001) or disease of the genitourinary system (OR = 1.39; 95%CI: 1.31–1.47; *p* < 0.001) had much higher odds of COVID 19–related risk of in-hospital death. The presence of at least one comorbidity more than doubled the COVID 19–related risk of in-hospital death (OR = 2.23; 95%CI: 2.14–2.32; *p* < 0.01). The following predictors of admission to ICU were found in multivariable analysis: age over 60 years (OR: 2.03; 95%CI: 1.90–2.16), male sex (OR: 1.79; 95%CI: 1.69–1.89), presence of at least one cardiovascular disease (OR: 1.26; 95%CI: 1.19–1.34), presence of at least one endocrine, nutritional and metabolic disease (OR: 1.17; 95%CI: 1.07–1.28).

## 1. Introduction

The severe acute respiratory syndrome coronavirus 2 (SARS-CoV-2) is a global public health concern because of its higher transmission around the world [1,2]. The estimated reproductive number is 2.87 (95%CI: 2.39–3.44) [3]. SARS-CoV-2 spreads mainly through human-to-human transmission via respiratory fluids generated when an infected person coughs, sneezes or speaks [2]. Moreover, airborne, fomite, fecal-oral, bloodborne, mother-to-child transmission have been reported [4]. SARS-CoV-2 is evolving towards higher transmissibility. As of July 2021, seven notable variants of SARS-CoV-2 has been reported [5].

SARS-CoV-2 causes Coronavirus Disease 2019 (COVID-19), a highly contagious infectious disease [1,2,3]. The most common COVID-19 symptoms are fever, cough, headache, fatigue, dyspnea and sputum [2,6]. The median incubation period is 4–5 days [1]. It is estimated that approximately a third of the people who are infected with the SARS-CoV-2 coronavirus do not develop noticeable symptoms at any point in time [7]. The illness severity can range from mild to critical. Of those people who develop noticeable symptoms, 81% develop mild to moderate symptoms (mild symptoms up to mild pneumonia), 14% develop severe symptoms such as dyspnea, hypoxia, or more than 50% lung involvement on imaging, and 5% of symptomatic COVID-19 cases is critical with respiratory failure, shock, or multiorgan system dysfunction [8]. The estimated global case fatality rate of COVID-19 is 2.2%, with markable differences by countries [9].

Several studies showed that older age and comorbidities such as cardiovascular disease, diabetes, chronic respiratory disease, hypertension, obesity and cancer are risk factors for a severe course of illness, complications and death from COVID-19 [10,11]. Moreover, tobacco use and exposure to air pollution contributes to the increased health risk of COVID-19 [12,13]. In the cohort of 2215 US adults with COVID-19 who were admitted to intensive care units, older age, male sex, morbid obesity, coronary artery disease, cancer, acute organ dysfunction and admission to a hospital with fewer intensive care unit beds were factors associated with COVID-19 death [14]. Among COVID-19 patients in Sweden, hypertension, type 2 diabetes mellitus, chronic renal failure, asthma, obesity, being a solid organ transplant recipient and immunosuppressant medications were independent risk factors of ICU admission [15]. A retrospective observational study using the Hospital Episode Statistics administrative dataset in England showed significant changes in COVID-19 in-hospital mortality in hospitalized adults over the first seven months of the pandemic (March–September 2020) [16]. Due to the health inequities between the countries and regions, differences in the healthcare systems and access to healthcare, detailed, national data on patient characteristics, treatment and outcomes of COVID-19 are needed to inform decision-makers and healthcare professionals about resource allocation and healthcare capacity.

Poland is the fifth most populated country in the European Union (EU). In 2020, the population of Poland amounted to around 38 million inhabitants. The first COVID-19 case was reported on 4 March 2020 [17]. As of 1 July 2021, 2.88 million COVID-19 cases and 75 thousand COVID-19-related deaths were reported in Poland [9]. Epidemiological data showed that there were three waves of the COVID-19 pandemic in Poland: March-June 2020, October–December 2020, and March–May 2021. During the first wave of the COVID-19 pandemic, dedicated COVID-19 centers were organized for hospitalization of COVID-19 patients, which expanded the resources already available in infectious and observation-infectious hospitals and wards [18]. During the second wave of the COVID-19 pandemic, in addition to hospitals dedicated to COVID-19, general-profile hospitals were transforming selected wards (most often internal medicine wards) into wards dedicated to COVID-19 patients. In addition, a temporary COVID-19 hospitals were created in stadiums and congress centers [9,18]. Each patient with COVID-19, in a health condition eligible for hospitalization, had the possibility of free hospitalization under the public healthcare system.

The aim of this study was to characterize patients hospitalized with COVID-19 between March and December 2020 as well as to identify factors associated with COVID-19-related risk of in-hospital death.

## 2. Materials and Methods

### 2.1. Data Source

This retrospective analysis was based on data from hospital discharge reports on COVID-19 patients hospitalized in Poland between March and December 2020. Hospital discharge reports are collected by the National Institute of Public Health as a part of the population-based hospital morbidity study [19]. According to Polish law, all hospitals (both public and private, except the psychiatric facilities) are obligated to report hospitalization data (discharge reports) according to one specific template. Discharge reports are anonymous and include data on gender, age, place of residence, hospital admission and discharge data, the primary and secondary diagnosis according to the 10th revision of the International Statistical Classification of Diseases and Related Health Problems (ICD-10), the outcome of hospitalization (death or survival). Data on medical conditions are filled by the physicians based on the ICD-10 medical classification.

### 2.2. COVID-19 Reporting and Outcome

COVID-19 patients were identified through the ICD-10 discharge diagnosis code of U07.1 (COVID-19, virus identified), which is attributable to the laboratory-confirmed COVID-19 diagnosis or U07.2 (COVID-19, virus not identified), which is attributable to the clinical or epidemiological diagnosis of COVID-19 (no conclusive laboratory confirmation/no laboratory confirmation available). Both confirmed cases of COVID-19, as well as probable cases of COVID-19 were analyzed because the COVID-19 case definition for epidemiological supervisions of SARS-CoV-2 coronavirus infection [20] consider the laboratory criteria (confirmed COVID-19 case), as well as clinical criteria (symptoms typical for COVID-19) and radiological criteria (CT-images of the lungs characteristic for COVID-19). The similar approach was applied in previous papers on COVID 19-related risk of in hospital death, where the data source were medical registers [21,22]. In Poland, COVID-19 death is defined in line with the European Centre for Disease Prevention and Control guidelines, which ensure the comparability of data between the EU countries [23].

The primary outcome was the discharge status of hospitalization: death or survival. Moreover, a separated analysis was carried out for transfer to the intensive care unit (ICU).

Comorbidities were grouped into categories based on the ICD-10 medical classification and included: endocrine, nutritional and metabolic disease (E00-90), cardiovascular diseases (I00-I99), disease of the genitourinary system (N00-99). It was assumed that viral pneumonia and acute respiratory distress syndrome (the most common diseases of the respiratory system in the analyzed group) are complications of COVID-19, and due to this fact only chronic obstructive pulmonary disease (COPD; J44) was included as a chronic respiratory disease that may affect the COVID 19–related risk of in hospital death.

### 2.3. Statistical Analysis

The data were analysed with SPSS version 28 (IBM, Armonk, NY, USA). Normality of distributions of continuous variables was assessed by the Shapiro–Wilk test. Statistical significance of differences between mean values of continuous variables was analysed by the independent samples *t*-test or if the assumptions for this were not met, the Mann–Whitney U test was used. The distribution of categorical variables was shown by counts and percentages. Statistical testing to compare categorical variables was completed using the independent samples chi-square test.

Associations between age, sex, and comorbidities with outcome of hospitalization (death or survival) were analyzed using the logistic regression analyses. Moreover, logistic regression analyses were used to assess the associations between age, sex and comorbidities with admission to the ICU.

Age, sex, presence of at least one cardiovascular disease (I00-I99), COPD, presence of at least one endocrine, nutritional and metabolic disease (E00-99) and presence of at least one disease of the genitourinary system (N00-99) were considered as independent variables. In univariate logistic regression analyses, all variables were considered separately. Multivariate logistic regression analyses included all the variables significantly associated with (1) the COVID 19–related risk of in-hospital death, as well as (2) with the risk of admission to the intensive care unit. The strength of association was measured by the odds ratio (OR) and 95% confidence intervals (CI). Statistical inference was based on the criterion *p* < 0.05.

### 2.4. Ethics

This study was carried out in accordance with the principles expressed in the Declaration of Helsinki. Epidemiological reports are anonymous and prevent identification of any individual study subject by the research team at any stage of the study. The study protocol was reviewed and approved by the Ethics Review Board at the Central Clinical Hospital of the Ministry of the Interior and Administration in Warsaw, Warsaw, Poland (approval number 45/2021). As we were working on secondary, anonymous data collected at the province level, written informed consent to participate in the study was not required.

## 3. Results

### 3.1. Characteristics of 116,539 Patients Hospitalized with COVID-19 in 2020

**Figure 1:**
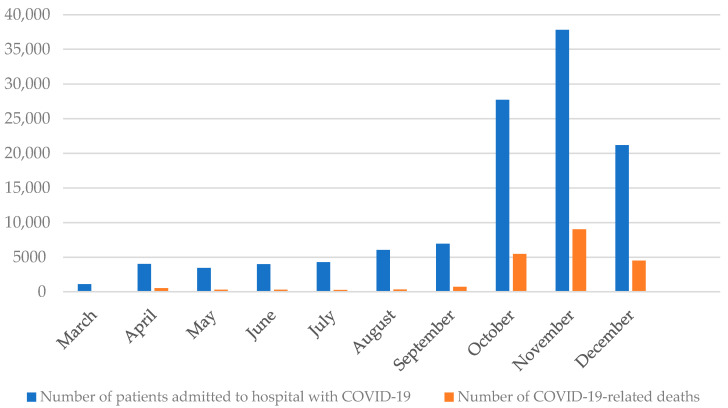
Number of patients admitted to hospital with COVID-19 and deaths in patients hospitalized with COVID-19, Poland, March–December 2020.

In 2020, a total of 116,539 patients were admitted to hospital with COVID-19 and 21,490 (18.4%) COVID-19-related deaths were reported. During the first wave of the COVID-19 pandemic in Poland (March–June 2020), a total of 12,572 patients were admitted to hospital with COVID-19, and 1041 COVID-19-related deaths were reported (frequency of COVID-19-related deaths: 8.3%). During the second wave of the COVID-19 pandemic (September–December 2020), a total of 93,649 patients were admitted to hospital with COVID-19, and 19,688 COVID-19-related deaths were reported (frequency of COVID-19-related deaths: 21.0%). A total of 10,318 patients were admitted to hospital with COVID-19 during the summer (July–August 2020) and 581 COVID-19-related deaths were reported. The highest frequency of COVID-19-related deaths was observed in November (23.8%) and the lowest in August (5.3%). Details are presented in Figure 1.

Characteristics of the study population is presented in Table 1. Out of 116,539 patients hospitalized with COVID-19 in 2020, 80.2% stayed in only one ward during hospitalization, 17.2% stayed in two wards, 2.2% stayed in three wards, and 0.3% stayed in four wards and 118 patients (0.01%) stayed in five wards during hospitalization. The most common first-admission ward was the emergency department (31.2%) and almost every fourth patient (24.1%) was admitted to the internal medicine ward. Out of all 116,539 patients hospitalized with COVID-19, 5734 patients (4.9%) were admitted to the ICU during hospitalization. Out of patients who stayed in two wards (*n* = 20,042), 31.2% were transferred from the first-admission ward to the internal medicine ward. Overall, the mean duration of hospitalization was 8.8 ± 9.4 days.

### 3.2. COVID 19–Related Risk of in-Hospital Death

The results of the univariate and multivariate regression analyses are presented in Table 2. Several characteristics, such as age, sex and presence of comorbidities were significantly associated with COVID 19–related risk of in-hospital death (Table 2). Patients over 60 years of age (OR = 7.74; 95%CI: 7.37–8.12; *p* < 0.001), men (OR = 1.42; 95%CI: 1.38–1.47; *p* < 0.001) as well as those with cardiovascular diseases (OR = 1.51; 95%CI: 1.46–1.56; *p* < 0.001) or disease of the genitourinary system (OR = 1.39; 95%CI: 1.31–1.47; *p* < 0.001) had much higher odds of COVID 19–related risk of in-hospital death. The impact of comorbidities on COVID 19-related risk of in-hospital death (Table 2). When controlled for age and sex (Table 3), the presence of at least one comorbidity more than doubled odds of COVID 19-related risk of in-hospital death (OR = 2.23; 95%CI: 2.14–2.32; *p* < 0.01).

### 3.3. Predictors of Admission to the Intensive Care Unit (ICU)

Five predictors of admission to ICU were found in multivariable analysis (Table 4), including age over 60 years (OR: 2.03; 95%CI: 1.90–2.16), male sex (OR: 1.79; 95%CI: 1.69–1.89), presence of at least one cardiovascular disease (OR: 1.26; 95%CI: 1.19–1.34), presence of at least one endocrine, nutritional and metabolic disease (OR: 1.17; 95%CI: 1.07–1.28) and COPD (OR: 0.64; 95%CI: 0.50–0.84) (*p* < 0.001).

When controlled for age and sex (Table 5), the presence of at least one comorbidity among patients with COVID-19 more than six-fold increased odds of risk of in-hospital the risk of admission to the intensive care unit (OR = 6.61; 95%CI: 5.96–7.33; *p* < 0.001).

## 4. Discussion

To the best of the author’s knowledge, this is the most comprehensive study on characteristics and clinical outcomes of patients hospitalized with COVID-19 during the first two waves of COVID-19 pandemic (March–December 2020) in Poland. Moreover, this study includes data on more than 110,000 patients hospitalized with COVID-19, so this is one of the largest studies published so far. This study showed that older age, male sex and presence of comorbidities are associated with COVID 19-related risk of in-hospital death. We observed that 4.9% of patients with COVID-19 were admitted to the ICU. Moreover, the results of our study showed that the cumulative in-hospital COVID-19 fatality rate was 18.4% over the period from March to December 2020. This study showed that older age, male sex and presence of comorbidities are associated with COVID 19-related risk of in-hospital death as well as the risk of admission to the intensive care unit.

In 2020, there were two waves of the COVID-19 pandemic in Poland. More than 80% of hospitalized COVID-19 patients were admitted to hospital during the second wave of COVID-19 pandemic (between September and December 2020). This observation is in line with the dynamics of COVID-19 pandemic in Poland [9,17]. In all EU countries, the autumn wave of the COVID-19 pandemic was much more dangerous than the spring one [9]. In Poland, the highest daily number of new COVID-19 were observed in November (on average 20,000 new COVID-19 per day). The high COVID-19 burden in November led to the fact that in November there were the highest number of hospital admissions due to COVID-19 (over 37.7 thousand people admitted to hospital) as well as the highest frequency of COVID-19-related in-hospital deaths (23.8%). Most of the patients (80.2%) stayed in only one ward during hospitalization, and the most common first-admission ward was the emergency department (31.2%) which suggests that a significant group of patients were people with a sudden deterioration of health, who were discharged home after a short hospitalization and education on checking COVID-19 symptoms and reporting information.

This study presents detailed characteristics of patients hospitalized with COVID-19. We observed that 60% of patients hospitalized with COVID-19 were over 60 years of age. Children and adolescent up to 19 years of age accounted for less than 5% of all patients hospitalized with COVID-19. Patients who died during hospitalization were significantly older compared to those who survive. The most common comorbidities observed among patients with COVID-19 were cardiovascular diseases. Cardiovascular diseases, endocrine, nutritional and metabolic diseases and COPD, as well as diseases of the genitourinary system were more frequently observed among those who died compared to those who survived. Our study is in line with previous studies on comorbidities among patients with COVID-19, however, the frequency of subsequent diseases differs across the studies due to different methodologies and populations [10,11,16,21,22]. In our study, 65% of patients hospitalized with COVID-19 had at least one comorbidity. The results of meta-analysis based on 29,096 COVID-19 cases showed, that 40.8% had comorbidities, while among fatal cases the frequency of comorbidities was 74.4% [24]. In the cohort of 2215 US adults with COVID-19, who were admitted to intensive care units, 78.5% had at least 1 chronic comorbidity [14]. In our study 83.1% patients with COVID-19 who died during hospitalization had at least 1 comorbidity that is comparable to the study carried out in the cohort of US adults with COVID-19, who were admitted to the ICU [14].

In this study, age over 60 years, men as well as those with cardiovascular diseases or disease of the genitourinary system (mostly chronic kidney disease) had much higher odds of COVID 19–related risk of in-hospital death. Age has the highest influence on COVID 19-related risk of in-hospital death. This observation is in line with epidemiological data on COVID-19 mortality in different age groups [21,22,24]. A study in a group of 2830 patients hospitalized with COVID-19 in one of the administrative regions in Poland (Silesia Voivodeship) showed that COVID-19 deaths were associated with male sex (OR: 1.52; 95%CI: 1.17–1.96), older age (OR: 6.11; 95%CI: 4.5–8.31), and the presence of 2 (OR: 3.05; 95%CI: 2.07–4.51) or ≥3 coexisting diseases (OR: 4.78; 95%CI: 3.52–6.49) [25]. In our study when controlled by age and sex, the presence of at least one comorbidity more than doubled the COVID 19–related risk of in-hospital death. The differences may result from the fact that the study from Silesia Voivodeship [25] was limited to the first wave of COVID-19 pandemic (March–June 2020) and our study includes data covering the entire 2020 (two waves of COVID-19 pandemic).

Moreover, this study is the first nationwide study assessing the risk of admission to the ICU among patients with COVID-19 in Poland. Age over 60 years, male sex, presence of at least one cardiovascular disease and presence of at least one endocrine, nutritional and metabolic disease had increased risk of ICU admission among patients with COVID-19. The presence of at least one comorbidity has the highest influence on the risk of ICU admission. This observation is different from that observed in the case of COVID 19–related risk of in-hospital death when age has the highest impact on the outcome of hospitalization. In this study, patients with COPD had a lower risk of ICU admission, but this observation should be interpreted carefully. For many years, there has been a debate in Poland about the underdiagnosis of COPD. Epidemiological data suggest that even up to 80% of COPD cases in Poland may be underdiagnosed [26]. We can hypothesize that the association between COPD and risk of ICU admission among COVID-19 patients may result from the COPD underdiagnosis in Poland.

This study has several practical implications. Our study provides comprehensive characteristics of patients hospitalized with COVID-19 in Poland. These data may be used by the epidemiologist to forecast the dynamics of the COVID-19 in Poland as well as in Central and Eastern Europe (CEE). Moreover, data presented in our study may be a basis for international comparisons on the effectiveness of anti-epidemic measures implemented among the EU countries. Factors associated with COVID 19–related risk of in-hospital death, as well as the risk of ICU admission, may be used by policymakers to allocate resources necessary to combat COVID-19 pandemic as well as to prepare healthcare capacity in response to the increasing number of COVID-19 cases. Furthermore, based on our study, healthcare professionals can identify high-risk groups for COVID-19, as well as to identify patients with a higher risk of admission to ICU based on the demographic data and underlying conditions that may be used during the selection of treatment methods and patient management.

This study has several limitations. First, this study is a retrospective, secondary analysis of epidemiological data. However, more than 110,000 discharge reports on patients hospitalized with COVID-19 from all administrative regions in Poland were analyzed. Secondly, the format of discharge reports is limited to one specific template and does not allow the identification of some risk factors like tobacco use as well as COVID-19 treatment (drugs) applied in individual patients with COVID-19. Thirdly, in this study, both laboratory-confirmed COVID-19 cases, as well as COVID-19 cases based on the clinical or epidemiological diagnosis, were included. However, according to the World Health Organization, the case definition of COVID-19 cases may be adapted to the local epidemiological situation and certain epidemic-related factors such as testing capacity, which is in line with the methods applied in our study.

## 5. Conclusions

More than half of patients hospitalized with COVID-19 in Poland were over 60 years of age, and children accounted for less than 5% of all patients hospitalized with COVID-19. The cumulative in-hospital COVID-19 fatality rate was 18.4% over the period from March to December 2020. COVID 19-related risk of in-hospital death was associated independently with sex, age, and the presence of cardiovascular diseases and diseases of the genitourinary system. Approximately 5% of patients hospitalized with COVID-19 were admitted to the ICU, wherein the risk of ICU admission was associated with age, sex and the presence of cardiovascular diseases or endocrine, nutritional and metabolic disease.

## Figures and Tables

**Table 1:** Characteristics of 116,539 patients hospitalized with COVID-19 by sex, Poland, March–December 2020.

Variable	Total Sample			*p*	Women			*p*	Men			*p*
	Overall *n* (%)	Fatal *n* (%)	Non-Fatal *n* (%)		Overall *n* (%)	Fatal *n* (%)	Non-Fatal *n* (%)		Overall *n* (%)	Fatal *n* (%)	Non-Fatal *n* (%)	
*n* (%)	116,539 (100%)	21,490 (18.4%)	95,049 (81.6%)		55,624 (47.7%)	8719 (15.7%)	46,905 (84.3%)		60,915 (52.3%)	12,771 (21.0%)	48,144 (79.0%)	
Age (years)												
0–9	3725 (3.2%)	2 (0.0%)	3723 (3.9%)	<0.0001	1609 (2.9%)	0 (0.0%)	1609 (3.4%)	<0.0001	2116 (3.5%)	2 (0.0%)	2114 (4.4%)	<0.0001
10–19	2154 (1.8%)	6 (0.0%)	2148 (2.3%)		1062 (1.9%)	0 (0.0%)	1062 (2.3%)		1092 (1.8%)	6 (0.0%)	1086 (2.3%)	
20–29	5710 (4.9%)	39 (0.2%)	5671 (6.0%)		3262 (5.9%)	20 (0.2%)	3242 (6.9%)		2448 (4.0%)	19 (0.1%)	2429 (5.0%)	
30–39	8794 (7.5%)	152 (0.7%)	8642 (9.1%)		4463 (8.0%)	53 (0.6%)	4410 (9.4%)		4331 (7.1%)	99 (0.8%)	4232 (8.8%)	
40–49	11,147 (9.6%)	445 (2.1%)	10,702 (11.3%)		5244 (9.4%)	121 (1.4%)	5123 (10.9%)		5903 (9.7%)	324 (2.5%)	5579 (11.6%)	
50–59	15,248 (13.1%)	1364 (6.3%)	13,884 (14.6%)		7176 (12.9%)	402 (4.6%)	6774 (14.4%)		8072 (13.3%)	962 (7.5%)	7110 (14.8%)	
60–69	24,358 (20.9%)	4401 (20.5%)	19,957 (21.0%)		9859 (17.7%)	1333 (15.3%)	8526 (18.2%)		14,499 (23.8%)	3068 (24.0%)	11,431 (23.7%)	
70–79	23,678 (20.3%)	6490 (30.2%)	17,188 (18.1%)		10,749 (19.3%)	2506 (28.7%)	8243 (17.6%)		12,929 (21.2%)	3984 (31.2%)	8945 (18.6%)	
80+	21,725 (18.6%)	8591 (40.0%)	13,134 (13.8%)		12,200 (21.9%)	4284 (49.1%)	7916 (16.9%)		9525 (15.6%)	4307 (33.7%)	5218 (10.8%)	
Presence of coexisting diseases												
Yes	75,792 (65.0%)	17,852 (83.1%)	57,940 (61.0%)	<0.0001	34,448 (61.9%)	7220 (82.8%)	27,228 (58.0%)	<0.0001	41,344 (67.9%)	10,632 (83.3%)	30,712 (63.8%)	<0.0001
Presence of at least one cardiovascular disease (I00-I99)												
Yes	26,541 (22.8%)	7747 (36.0%)	18,794 (19.8%)	<0.0001	11,888 (21.4%)	3244 (37.2%)	8644 (18.4%)	<0.0001	14,653 (24.1%)	4503 (35.3%)	10,150 (21.1%)	<0.001
Presence of at least one endocrine, nutritional and metabolic disease (E00-99)												
Yes	10,165 (8.7%)	2115 (9.8%)	8050 (8.5%)	<0.001	5105 (9.2%)	985 (11.3%)	4120 (8.8%)	<0.001	5060 (8.3%)	1130 (8.8%)	3930 (8.2%)	0.01
Presence of at least one disease of the genitourinary system (N00-99)												
Yes	6280 (5.4%)	1808 (8.4%)	4472 (4.7%)	<0.001	2716 (4.9%)	718 (8.2%)	1998 (4.3%)	<0.001	3564 (5.9%)	1090 (8.5%)	2474 (5.1%)	<0.001
COPD												
Yes	1324 (1.1%)	353 (1.6%)	971 (1.0%)	<0.001	383 (0.7%)	81 (0.9%)	302 (0.6%)	0.003	941 (1.5%)	272 (2.1%)	669 (1.4%)	<0.001
Arterial hypertension												
Yes	12,227 (10.5%)	1993 (9.3%)	10,234 (10.8%)	<0.001	5754 (10.3%)	839 (9.6%)	4915 (10.5%)	0.02	6473 (10.6%)	1154 (9.0%)	5319 (11.0%)	<0.001
Diabetes mellitus												
Yes	6924 (5.9%)	1684 (7.8%)	5240 (5.5%)	<0.001	3140 (5.6%)	752 (8.6%)	2388 (5.1%)	<0.001	3874 (6.4%)	932 (7.3%)	2852 (5.9%)	<0.001

*p*—fatal vs. non-fatal COVID-19 cases.

**Table 2:** Odds ratios (OR) and 95% confidence intervals (CI) for COVID 19–related risk of in-hospital death to selected factors in a group of 116,539 patients hospitalized with COVID-19—Poland, March–December 2020.

Variable	Univariate Logistic Regression			Multivariate Logistic Regression ^a^		
	OR	95%CI	*p*	OR	95%CI	*p*
Age (years)						
<60	1.00	Reference		1.00	Reference	
≥60	8.50	8.10–8.91	<0.001	7.74	7.37–8.12	<0.001
Sex						
women	1.00	Reference		1.00	Reference	
men	1.43	1.39–1.47	<0.001	1.42	1.38–1.47	<0.001
Presence of at least one cardiovascular disease (I00-I99)						
No	1.00	Reference		1.00	Reference	
Yes	2.29	2.22–2.36	<0.001	1.51	1.46–1.56	<0.001
COPD						
Yes	1.00	Reference		1.00	Reference	
No	1.62	1.43–1.83	<0.01	0.88	0.78–1.00	0.05
Presence of at least one endocrine, nutritional and metabolic disease (E00-99)						
No	1.00	Reference		1.00	Reference	
Yes	1.18	1.12–1.24	<0.001	0.79	0.75–0.83	<0.001
Presence of at least one disease of the genitourinary system (N00-99)						
No	1.00	Reference		1.00	Reference	
Yes	1.86	1.76–1.97	<0.001	1.39	1.31–1.47	<0.001

^a^ Fully adjusted model including all statistically significant characteristics.

**Table 3:** The impact of comorbidities on COVID 19–related risk of in-hospital death controlled for the age and sex.

Variable	Multivariate Logistic Regression		
	OR	95%CI	*p*
Presence of at least one comorbidity			
No	1.00	Reference	
Yes	2.23	2.14–2.32	*p* < 0.001
Age (years)			
<60	1.00	Reference	
≥60	7.36	7.01–7.72	*p* < 0.001
Sex			
women	1.00	Reference	
men	1.40	1.36–1.45	*p* < 0.001

**Table 4:** Odds ratios (OR) and 95% confidence intervals (CI) for admission to the intensive care unit (ICU) to selected factors in a group of 116,539 patients hospitalized with COVID-19—Poland, March–December 2020.

Variable	Univariate Logistic Regression			Multivariate Logistic Regression ^a^		
	OR	95%CI	*p*	OR	95%CI	*p*
Age (years)						
<60	1.00	Reference		1.00	Reference	
≥60	2.17	2.04–2.31	<0.001	2.03	1.90–2.16	<0.001
Sex						
women	1.00	Reference		1.00	Reference	
men	1.80	1.70–1.90	<0.001	1.79	1.69–1.89	<0.001
Presence of at least one cardiovascular disease (I00-I99)						
No	1.00	Reference		1.00	Reference	
Yes	1.59	1.50–1.68	<0.001	1.26	1.19–1.34	<0.001
COPD						
Yes	1.00	Reference		1.00	Reference	
No	0.93	0.72–1.21	0.6	0.64	0.50–0.84	<0.001
Presence of at least one endocrine, nutritional and metabolic disease (E00-99)						
No	1.00	Reference		1.00	Reference	
Yes	1.40	1.27–1.50	<0.001	1.17	1.07–1.28	<0.001
Presence of at least one disease of the genitourinary system (N00-99)						
No	1.00	Reference		1.00	Reference	
Yes	1.26	1.13–1.41	<0.001	1.04	0.93–1.16	0.5

^a^ Fully adjusted model including all statistically significant characteristics.

**Table 5:** The impact of comorbidities on risk of admission to the intensive care unit (ICU) controlled for the age and sex.

Variable	Multivariate Logistic Regression		
	OR	95%CI	*p*
Presence of at least one comorbidity			
No	1.00	Reference	
Yes	6.61	5.96–7.33	*p* < 0.001
Age (years)			
<60	1.00	Reference	
≥60	1.61	1.51–1.72	*p* < 0.001
Sex			
women	1.00	Reference	
men	1.69	1.60–1.79	*p* < 0.001

## Data Availability

The datasets generated during and/or analysed during the current 350 study are available from the corresponding author on reasonable request.
